# The impact of rare and low-frequency genetic variants in common variable immunodeficiency (CVID)

**DOI:** 10.1038/s41598-021-87898-1

**Published:** 2021-04-15

**Authors:** Atil Bisgin, Ozge Sonmezler, Ibrahim Boga, Mustafa Yilmaz

**Affiliations:** 1 Department of Medical Genetics, Faculty of Medicine, Balcali Hospital and Clinics, Cukurova University, Adana, Turkey; 2 Cukurova University AGENTEM (Adana Genetic Diseases Diagnosis and Treatment Center), Adana, Turkey; 3 Division of Pediatric Allergy and Immunology, Faculty of Medicine, Balcali Hospital and Clinics, Cukurova University, Adana, Turkey

**Keywords:** Cancer genetics, Cancer genomics, Clinical genetics, Sequencing, Immunological disorders, Immunological disorders

## Abstract

Next Generation Sequencing (NGS) has uncovered hundreds of common and rare genetic variants involved in complex and rare diseases including immune deficiencies in both an autosomal recessive and autosomal dominant pattern. These rare variants however, cannot be classified clinically, and common variants only marginally contribute to disease susceptibility. In this study, we evaluated the multi-gene panel results of *Common Variable Immunodeficiency* (CVID) patients and argue that rare variants located in different genes play a more prominent role in disease susceptibility and/or etiology. We performed NGS on DNA extracted from the peripheral blood leukocytes from 103 patients using a panel of 19 CVID-related genes: *CARD11, CD19, CD81, ICOS, CTLA4, CXCR4, GATA2, CR2, IRF2BP2, MOGS, MS4A1, NFKB1, NFKB2, PLCG2, TNFRSF13B, TNFRSF13C, TNFSF12, TRNT1* and *TTC37.* Detected variants were evaluated and classified based on their impact, pathogenicity classification and population frequency as well as the frequency within our study group. NGS revealed 112 different (a total of 227) variants with under 10% population frequency in 103 patients of which 22(19.6%) were classified as benign, 29(25.9%) were classified as likely benign, 4(3.6%) were classified as likely pathogenic and 2(1.8%) were classified as pathogenic. Moreover, 55(49.1%) of the variants were classified as variants of uncertain significance. We also observed different variant frequencies when compared to population frequency databases. Case–control data is not sufficient to unravel the genetic etiology of immune deficiencies. Thus, it is important to understand the incidence of co-occurrence of two or more rare variants to aid in illuminating their potential roles in the pathogenesis of immune deficiencies.

## Introduction

Common variable immunodeficiencies (CVIDs) are a genetically heterogeneous group of disorders which have familial and sporadic genetic background in both an autosomal recessive and autosomal dominant pattern^1^. They are characterized by hypogammaglobulinemia, insufficiency of specific antibody production and recurring infections. The European Society for Immunodeficiency (ESID) and the Pan American Group for Immunodeficiency’s definition of CVID are the most cited description of the disease. Patients need two of the following criteria for assignment as “probable CVID”: greater than 2 years of age; immunoglobulin G (IgG) and immunoglobulin A (IgA) less than 2 standard deviations from the mean for age; either absent isohemaglutinin or absent vaccine responses;’ and no other defined causes of hypogammaglobulinemia^2,3^. It has been shown that genetic factors are one of the causes of the onset and progression of CVID. Monogenic defects meet the criteria for a small part of CVID diagnoses, however monogenicity is not applicable for all cases because of the disease’s heterogeneity and the inheritance patterns of disease related genes. More complex genetic scenarios are in consideration for the disease’s possible oligogenic background^4^. Some studies also showed that epigenetic factors such as altered demethylation and hypermethylation play a significant role in pathogenic mechanism and clinical manifestations of the disease^5,6^.

Genome-Wide Association (GWAS) studies that are followed by Next Generation Sequencing (NGS) have been shown to be the most useful methods for discerning the genetic underpinnings of these immunopathologies and for developing improved genetic testing^4,7–10^. However, dominancy of the genes and the mutations can also be determinant for disease pathogenesis. As a result of recent findings with regard to disease heterogeneity and polygenicity, NGS technologies are gaining in popularity for CVID diagnostics^11^. Accordingly, utilization of multi-gene panels for diagnostics and gene discovery has increased for CVID leading to increase in precision. In our previous study, we have highlighted the clinical utility of molecular testing with immune phenotyping. However, there is still a challenge in the field of genomics in that when potential novel genes/mutations or none are detected. Still, multigene panel testing in the diagnosis of clinically heterogeneous diseases such as primary immune deficiency disorders (PIDs) became most successful diagnostic tool but only in hands of experienced centers for detection of clinically relevant variants even though the underestimated rare variants that have to be correlated with the clinical phenotype^12^. We thus emphasized that evaluating variants with low population frequencies is important to understand disease pathogenicity.

Replacement therapies and/or immune supportive treatments are the most common approaches for CVID patients, however it is not effective for all individuals, due to the fact that the disease presents differently among patients because of the underlying genetic heterogeneity. Using data accumulated through the use of NGS technologies and enhanced genetic testing, we suggest that some combinations of multiple low frequency genetic variants may have a cumulative effect leading to disease onset and progression. Such data could be utilized to help determine the most effective immunoglobulin replacement therapy option during the clinical decision-making process.

CVID prevalence varies significantly among countries and populations. Together with its polygenicity, distinctive prevalence and disease heterogeneity, evaluating CVID in terms of the common disease-common variant (CDCV) hypothesis is not useful in many cases^13^. When considering the increased frequency of rare diseases in our community, immunodeficiencies and CVID specifically, it is crucial to study the disease’s etiology and immunopathology in terms of the common disease-rare variant (CDRV) hypothesis^14^.

Although there are CVID-associated alleles that have been defined in literature, variant classification plays an important role in disease and patient management. Pathogenicity classifications of variants are made based on multiple criteria including, but not necessarily limited to; population data; position of the alteration; in silico prediction scores, and submitted cases^15^. Pathogenic and likely pathogenic variants when detected explain the disease phenotype the majority of the time. However, the genotype–phenotype evaluation is harder to perform in some cases when there are only variants of uncertain significance, benign and likely benign changes considering the impact of detected variants may differ in each patient and as the disease progress. We believe that variants of uncertain significance might have a greater effect on the disease’s etiology and progress, and should be further investigated for clinical interpretation. Additionally, they should be examined in consideration with less frequent variants for the possible population-specific cumulative effect on function.

## Methods

### Patient selection and sampling

**Figure 1:**
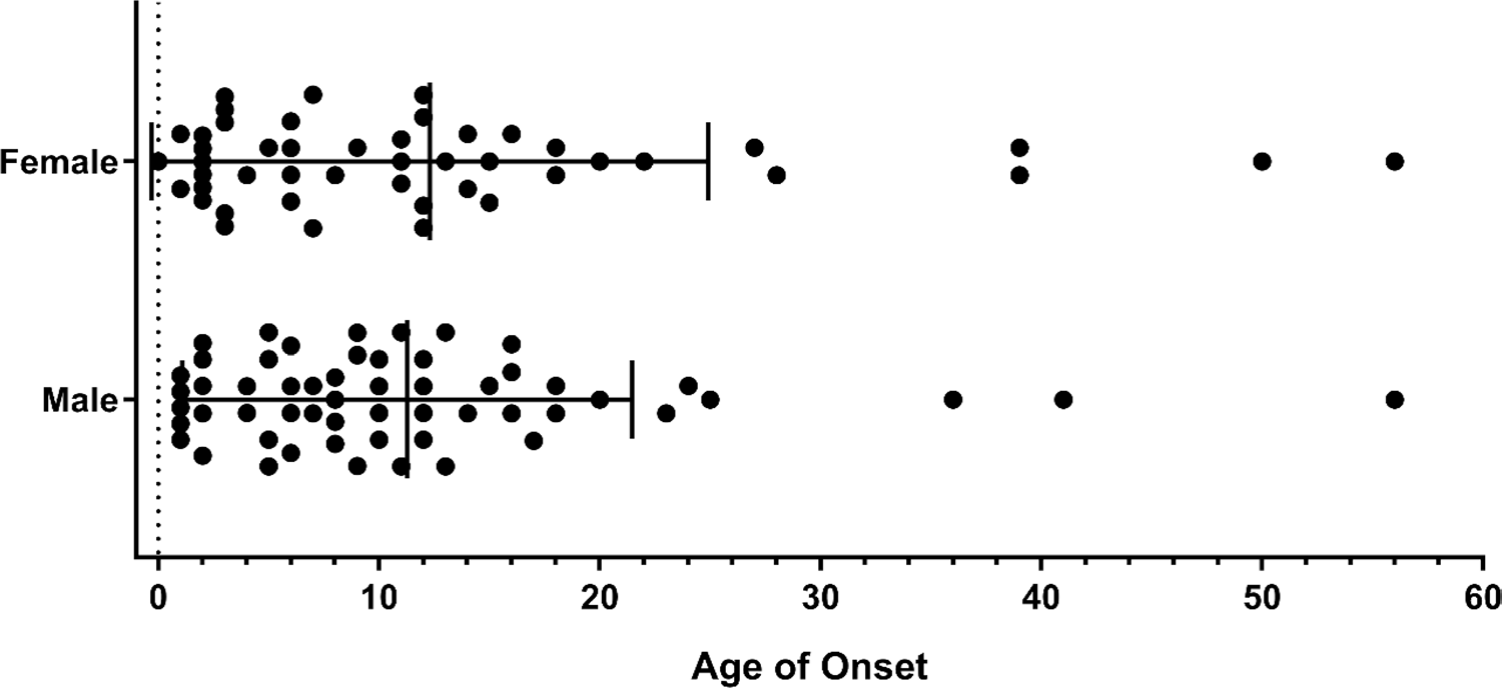
Age distribution of CVID onset by gender for the study group (The figure was created using GraphPad Prism version 7.0.0 for Windows, GraphPad Software, San Diego, California USA, www.graphpad.com).

Patients with a CVID presumptive diagnosis who met the ESID criteria for a CVID diagnosis were referred to Cukurova University AGENTEM for molecular genetic testing, and were selected retrospectively for this study. Informed consents were obtained for all the patients in accordance with the ethical standards of the institutional ethical committee (Cukurova University Faculty of Medicine Non-Invasive Clinical Research Ethics Commission) and the Helsinki declaration. The patients’ demographic information is presented as supplemental data (see Supplementary Table S1 online). The mean age for male patients was 11.3; for female patients it was 12.3. The overall mean age of onset was 11.7 (Fig. 1). All the enrolled patients come from consanguineous families. Peripheral blood samples were collected from 103 patients. Genomic DNA was isolated via the QIAamp DNA Blood Mini Kit (Qiagen, Hilden, Germany), according to the manufacturer’s instructions. The quality of DNA samples was assessed with a Qubit Fluorimeter (Thermo Fisher Scientific, Waltham, MA, USA).

All the recruited patients were manifesting clinical findings and immunophenotying profiles of CVID who were also not characterized with more severe immune dysregulations and well identified syndromic diseases^16^. The study group consists of 103 patients’ that were diagnosed based on hypogammaglobulinemia, low level of specific antibodies and disturbances of cellular immunity reversing CD4/CD8 ratio. The clinical symptoms mainly included recurrent respiratory tract infections, pneumonias and sinusitis in older children. None of the patients had chronic thrombocytopenia while most had the autoimmune thrombocytopenia episodes.

### Next generation sequencing (NGS)

The Next Generation Sequencing workflow was performed to achieve a minimum of 300 × coverage on an Illumina MiSeq (California, United States) platform via a custom designed multi-gene panel (QIAseq Targeted DNA Custom Panels—CDHS-12337Z-606, QIAgen, Hilden, Germany) covering 19 disease-related genes: Caspase Recruitment Domain Family Member 11 (*CARD11*), *CD19*, *CD81*, Inducible T Cell Costimulator (*ICOS*), Cytotoxic T-Lymphocyte Associated Protein 4 (*CTLA4*), C-X-C Motif Chemokine Receptor 4 (*CXCR4*), GATA Binding Protein 2 (*GATA2*), (Complement C3d Receptor 2) *CR2*, Interferon Regulatory Factor 2 Binding Protein 2 (*IRF2BP2*), Mannosyl-Oligosaccharide Glucosidase (*MOGS*), Membrane Spanning 4-Domains A1 (*MS4A1*), Nuclear Factor Kappa B Subunit 1 (*NFKB1*), Nuclear Factor Kappa B Subunit 2 (*NFKB2*), Phospholipase C Gamma 2 (*PLCG2*), TNF Receptor Superfamily Member 13B (*TNFRSF13B*), TNF Receptor Superfamily Member 13C (*TNFRSF13C*), TNF Superfamily Member 12 (*TNFSF12*), TRNA Nucleotidyl Transferase 1 (*TRNT1*) and Tetratricopeptide Repeat Domain 37 (*TTC37*). It also included for all genes, all exons, introns, and 1 kb of the 5′ promoter regions and the 3′ UTRs. Inheritance patterns of genes for the CVIDs according to OMIM (Online Mendelian Inheritance in Man) were given in Table 1.

**Table 1:** Inheritance patterns of selected genes according to OMIM database.

Gene	MIM number	Inheritance
*CARD11*	607210	AD/AR
*CD19*	107265	AR
*CD81*	186845	AR
*CR2*	120650	AR
*CTLA4*	123890	AD
*CXCR4*	162643	AD
*GATA2*	137295	AD
*ICOS*	604558	AR
*IRF2BP2*	615332	AD
*MOGS*	601336	AR
*MS4A1*	112210	AR
*NFKB1*	164011	AD
*NFKB2*	164012	AD
*PLCG2*	600220	AD
*TNFRSF13B*	604907	AD/AR
*TNFRSF13C*	606269	AR
*TNFSF12*	602695	AD
*TRNT1*	612907	AR
*TTC37*	614589	AR

*AD* Autosomal dominant, *AR* Autosomal recessive.

Multi-gene panel was designed as a result of meta-analysis that was accordant with the OMIM entries of CVIDs and our population based phenotypic and genomic database^10,12,17^.

*CARD11* encodes a signaling scaffold protein that functions in upstream of the NF-κB pathway. Mutations in CARD11 gene leads to recurrent infections, B cell expansion, NF-κB hyper activation and T cell anergy^18,19^.

*CD19* express a cell surface protein in B lymphocytes. Mutations in this gene disrupt B-cell differentiation, cause impaired secretion of immunoglobulins and results with common variable immunodeficiency 3 (CVID3)^20^.

*CD81* encodes a surface glycoprotein known as tetraspin which is essential for clonal expansion of B cells and antibody production. Defects in this gene are associated with common variable immunodeficiency 6 (CVID6)^21^.

*ICOS* plays a significant role in cell–cell interaction, immune responses and efficient antibody secretion. Relation of recessively inherited mutations with CVID pathogenesis were reported^22,23^.

*CTLA4* belongs to immunoglobulin superfamily and encodes a protein that play role in T cell inhibition. CTLA4 is recently associated with both familial and non-familial forms of CVIDs and dysregulated immune responses^24,25^.

*CXCR4* takes place in orchestrating lymphocyte migration with chemokines and other chemokine receptors. Abnormal B and T cell trafficking and altered CXCR4 expressions were reported in CVID patients^26^.

*GATA2* deficient patients showed severe immunological features in parallel with senescent T cell phenotype and B cell lymphopenia. This gene is also reported more frequently in CVID pre-diagnosed patients in Mediterranean region populations^27,28^.

*CR2* gene is related to immune response lectin induced complement and hematopoietic cell lineage pathways. Genetic variations in this gene are correlated with common variable immunodeficiency 7 (CVID7)^29^.

*IRF2BP2* encodes a protein that act a part in transcriptional regulation of type 1 interferon. Keller et al. showed that the genetic alterations cause paired formation of B-cell plasmablasts and familial form of common variable immunodeficiency disorder (CVID14)^30^.

Patients with agammaglobulinemia or hypogammaglobulinemia can have immunoglobulin levels that look similar to CVID such as originating from *MOGS* as one of the common in the region^31^.

*MS4A1* gene Express a B-lymphocyte surface molecule. Homozygous mutations in this gene are related to common variable immunodeficiency 5 (CVID5) for damaging the development, differentiation and activation of B-cells^32^.

*NFKB1* and NFKB2 are associated with a numerous signal transduction pathways related to inflammation, immunity and differentiation. They play role in regulating immune response and acute phase reactions. Studies showed that NFKB1 and NFKB2 mutations are one of the most common monogenic causes of common variable immunodeficiency (CVID12 and CVID10)^33,34^.

PLCG2 gene’s association with antibody deficiency and auto-inflammation is known. It has a significant role in B cell activation by assisting the calcium mobilization. Hence, impairment of PLCG2 gene results in common immune dysregulation and CVID^35,36^.

*TNFRSF13B* [encoding transmembrane activator and CAML interactor (TACI)] gene involves in humoral immunity, B and T cell function. Mutations in this gene identified in both heterozygous, homozygous and/or compound heterozygous forms in patients with common variable immunodeficiency (CVID2). It has shown that TACI mutations have a significant proportion of regarding both disease susceptibility and pathogenesis^22,37–39^.

*TNFRSF13C* [B cell-activating factor receptor (BAFFR)] gene also plays a role in NF-κB pathway. It promotes B cell response and mature B cell survival with its ligand BAFF. Homozygous mutations were reported in patients with common variable immunodeficiency (CVID4) due to deficient IgG and IgM antibody production, pre-B cell transition cells increment while reduced class-switched memory B cells^40,41^.

Genetic changes in TNF protein family have been related to inherited forms CVID resulting impaired antibody response and recurrent infections. *TNFSF12* gene is also classified as disease-susceptibility gene for a humoral immunodeficiency^10,17,42^.

*TRNT1* encodes an enzyme necessary required for tRNA aminoacylation. Pathogenic variations in this gene cause mitochondrial translation, impaired clearance of tRNAs and impaired intracellular stress response eventually may end with B-cell immunodeficiency^43,44^.

*TTC37* gene is participated to cytosolic exosome’s large protein complex, and takes a part of inner cell abnormal mRNA degradation which has important role for cell growth. Genetic alterations in this gene were reported for specific antibody deficiency and defects in humoral memory^45,46^.

### QC of NGS data and bioinformatics analyses

Quality control parameters were checked for both sequencing and variant qualities via QCI-Analyze tool and the QCI-Interpret interface. Total yield, sequencing quality score, depth of coverage, quality score of variants, forward/reverse read balance, population and variant frequencies were assessed as primary variant analysis. For a variant to be included in our pathogenic analyses it had to have a population frequency of < 10%. The highest population frequency data was used as the population frequency threshold for each variant. Disease causing variants of more than 10% of population frequency were excluded from the study in order to examine the possible cumulative effects of rare variants. Next, the rare variants underwent a secondary classification according to their frequency in this study group. Lastly, variants were categorized based on their pathogenicity according to the American College of Medical Genetics (ACMG) criteria as pathogenic, likely pathogenic, variant of uncertain significance (VUS), likely benign and benign. In silico analysis tools including SIFT, B-SIFT, Polyphen-2, MutationTaster, BLOSUM, PROVEAN, CADD, DANN, GeneSplicer, PhyloP, MaxEntScan and QCI Inferred Activation were also used for the further examination of the VUS’s.

### Statistical analysis

ClinCalc (http://clincalc.com) was used to determine the post-hoc power of the study and to apply the Bonferroni correction. All statistical analyses were performed using the GraphPad Prism software (GraphPad Software, Inc. USA). Statistical significance was defined at p ≤ 0.05. Hardy Weinberg equilibrium analyses were performed for each variant identified. A modified version of the human genome (www.varsome.com) was used as the major allele population-specific reference. A confidence interval (CI) of 95% was used to estimate the precision of the odds ratio. A chi-square test was also used to test the frequencies of the alleles and genotypes^47^.

## Results

**Figure 2:**
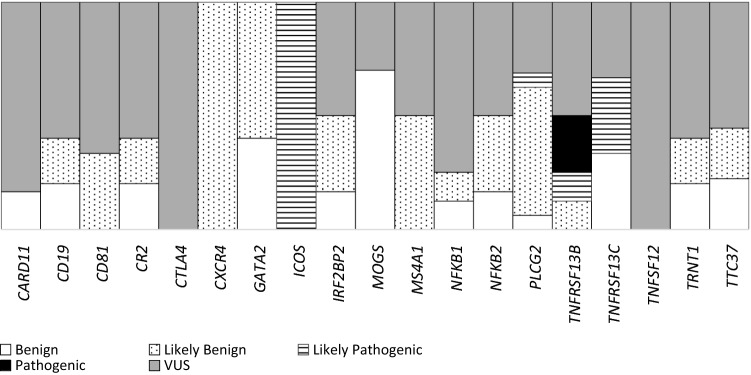
Distribution of the rare variants. Pathogenicity from benign to pathogenic are given accordingly with the ACMG criteria. The well-known pathogenic variants that have > 10% population frequency have been excluded while the pathogenic variants shown here are the ones with differential effects in different populations.

A total of 227 variants (112 total occurrences) with frequencies of less than 10% in the population were detected in 103 patients via NGS panel sequencing. Twenty-two (19.6%) of the 112 unique variants were classified as benign, 29 (25.9%) were classified as likely benign, 4 (3.9%) were classified as likely pathogenic (in *ICOS, PLCG2, TNFRSF13B* and *TNFRSF13C* genes) and 2 (1.8%) were classified as pathogenic (in *TNFRSF13B* gene). Additionally, 55 (49.1%) variants were classified as VUS. A majority of the rare variants were detected in the *PLCG2* and *CR2* genes. The gene distribution of the detected variants is presented in Fig. 2.

We observed different variant frequencies among our patient cohort as compared to population frequency databases including The Genome Aggregation Database and the Exome Sequencing Project. The observed variant frequencies for our patient cohort showed higher minor allel frequencies compared to global databases for: 2 of 2 pathogenic variants; 2 of 4 likely pathogenic variants; 46 of 55 VUSs; 20 of 29 likely benign variants and 3 of 22 benign variants. In addition, we detected 13 novel genetic changes in *CARD11* (n = 1), *CD19* (n = 1), *CR2* (n = 4), *CTLA4* (n = 1), *NFKB2* (n = 1), *TRNT1* (n = 2) and *TTC37* (n = 3) genes. Distribution of the detected variants and their study/population frequencies are shown in Supplementary Table S2 online.

Twelve of 103 patients (11.7%) had more than one variant of uncertain significance as listed in Supplementary Table S3. Among them, majority (n = 7, 58.3%) had VUS in *IRF2BP2* gene, and 25% (n = 3) had variations in either *CARD1, CR2, CD19,* or *NFKB2* genes. In addition, *TRNT1* and *NFKB1* genes were mutated in 2 (16.7%) patients while VUSs in *PLCG2, CD81, TNFRSF13B* and *TTC37* genes were detected in only one (8.3%) patient.

Thirteen (12.6%) patients had no rare variants detected. Eighteen (17.5%) patients had only one rare variant detected, while 33% of patients had 2 rare variants detected and the remaining 38 (36.9%) patients had more than 2 rare variants detected. Distribution of patients who carry 2 or more rare variants are given in Table 2. Clinical relevance and severity have been analyzed in relation to number of variants detected in each patient. However, due to the heterogeneous nature of CVIDs, and evidence for variation in the ratio of variants’ classifications, no difference was detected between the groups (See Supplementary Table S3).

**Table 2:** Distribution of patients with 2 or more rare variations.

Number of genetic changes	Patient distribution	
2 rare variants	33%	n = 34
3 rare variants	16.5%	n = 17
4 rare variants	13.6%	n = 14
5 rare variants	4.9%	n = 5
6 rare variants	1.9%	n = 2

## Discussion

A majority of the detected rare variants (49.1%) were classified as VUS. This indicates that a more thorough evaluation of these variants is necessary in order to be able to perform effective clinical interpretations. It is important to highlight these variants whose significance is uncertain in relation to the disease, because the etiology of many CVID cases is still unknown, and only through future research on candidate alleles will the field move forward.

Ninety of our CVID patients (87.4%) had at least one rare variant in a gene previously linked to an inherited immunodeficiency. We identified variant frequencies of many rare variants between our patient population and database values suggesting that some of these may be good candidates for future functional studies. The vast majority of the VUS, likely pathogenic and pathogenic variants (80.3%) were present at higher frequencies in our population than in public databases; whereas the majority of the 28 (54.9%) variants classified as likely benign and benign variants were found to be less frequent in our patient population than reported on a global scale.

Due to the fact that our study group only consisted of patients with clinical findings indicative of CVID, these results demonstrate the significance of proper NGS panel design for target diseases in combination with the correct patient selection criteria. Our results show that rare variant examinations are important as the majority of pathogenic and VUS-classified variants had elevated frequencies in our patient population.

Thus, based on our findings, not only should the CVCD hypothesis be evaluated but also the RVCD as well, and that in particular we emphasize that the cumulative effects of multiple rare and common variants should also be considered and that makes the diseased population specific studies more important as in our study.

In our analyses, seventy-two patients (69.9%) had more than 2 rare variants. We observed no differences between groups in the ratio of variants’ classifications (See Table 2 and Supplementary Table S3). Although the study cohort consists of clinically penetrant patients, a further prospective study including the patients with the family members is needed to understand the possible clinical impact of coexisting rare variants. Unique roles of rare variants in the genetics of such a complex disease of CVIDs have distinctive features. However, due to the lower linkage, the basis of such clinical background effects has not been specifically studied.

We also observed that twelve patients (11.7%) had more than 2 rare variants with uncertain significance. Incidence of *IRF2BP2* gene variations (7/12, 58.3%) formed the majority followed by *CARD1, CR2, CD19, NFKB2, TRNT1, NFKB1, PLCG2, CD81, TNFRSF13B and TTC37* genes*.* However, population based whole genome studies together should be performed for precise data interpretation of VUSs.

The diverse findings of variant numbers detected in CVID patients need to be interpreted. However, this study is still the first characterized composition of variant and allelic frequency based subsets in CVID patients. Our prospective long-term study of clinical evaluation will become more important to clarify the disease perpetuation.

Besides all, one of the limitations of our study is not being able to eliminate other possible monogenic defects which is why a comparison analysis with the exome data sets should be established for further investigation for a more comprehensive approach for considering inborn errors of immunity. Also, a population specific study is another necessity to reveal possible founder affects. In order to do that, pathway analysis for each rare variant; and a network analysis for the patients who carry two or more rare variants should be practiced.

Functional validation is the step ahead for understanding the significance of mutations. It is especially important to further examine the gene–gene and protein–protein interactions in the cases with oligogenic background for possible epistasis. In silico analysis tools provide predictive outcomes. However, it may be problematic to enlighten the impacts of heterozygous variations in-vivo or ex-vivo if more than one gene is affected^8^.

Despite the immunodeficiencies were reported in different ethnic groups, there are not enough information about the disease prevalence. Although the whole exome and genome databases provide large datasets, specific populations or the groups with individuals of mixed background may be underrepresented. Hence, we cannot exclude the possibility that disease prevalence differs between some ethnic groups. There is also a chance of the genotype prevalence in gnomAD is inflated due to the analysis assuming each variant is found on a different allel. So that the case–control data is not sufficient in all cases to unravel the genetic etiology of immune deficiencies. Thus, it is important to understand the incidences of two or more rare variants’ coexistence for the possible synergistic roles they play in the pathogenesis of immune deficiencies. Additionally, the population specific data may play a role in the evaluation or re-assessment of the detected variants, especially the VUS. Therefore, further corresponding studies to establish local and/or national allele frequency databases has become a necessity.

## Supplementary information

Supplementary information 1.

Supplementary information 2.

Supplementary information 3.
