# Supplementary material for: The impact of rare and low-frequency genetic variants in common variable immunodeficiency (CVID)
Source: Sci Rep. 2021 Apr 15;11:8308. doi: 10.1038/s41598-021-87898-1 (PMC8050305; doi:10.1038/s41598-021-87898-1)
Supplement: Supplementary file 1 — Supplementary information 1. [file 41598_2021_87898_MOESM1_ESM.pdf]

The Impact of Rare and Low-Frequency Genetic Variants in Common Variable Immunodeficiency (CVID)

Atil Bisgin[1, 2], Ozge Sonmezler[2], Ibrahim Boga[1, 2], Mustafa Yilmaz[3]

Supplementary Table S1. Demographic information of the patients.

Patient number	Gender	Date of birth	Age of onset	Patient number	Gender	Date of birth	Age of onset
**1**	F	2005	11	**53**	M	2003	13
**2**	F	2014	2	**54**	F	2013	5
**3**	M	2010	6	**55**	F	1979	39
**4**	F	2015	1	**56**	M	2017	1
**5**	F	2000	16	**57**	F	2007	11
**6**	F	2014	2	**58**	M	2002	16
**7**	F	2015	1	**59**	M	2002	16
**8**	F	2014	2	**60**	M	2004	14
**9**	M	2005	11	**61**	M	2012	6
**10**	M	2008	9	**62**	F	2006	12
**11**	F	2003	13	**63**	F	2016	2
**12**	M	2010	6	**64**	M	2007	11
**13**	M	2008	8	**65**	M	1962	56
**14**	F	2016	0	**66**	F	2003	15
**15**	M	2007	9	**67**	F	1990	28
**16**	M	2005	12	**68**	F	1962	56
**17**	M	2007	10	**69**	F	2012	6
**18**	M	2015	2	**70**	F	1968	50
**19**	M	2010	7	**71**	F	2006	12
**20**	F	2010	7	**72**	M	2013	5
**21**	M	2007	10	**73**	F	1991	27
**22**	M	2001	16	**74**	F	2012	6
**23**	F	2013	4	**75**	M	2010	8
**24**	M	2016	1	**76**	M	2013	5
**25**	M	1992	25	**77**	M	1994	24
**26**	M	2015	2	**78**	M	2016	2
**27**	M	2011	6	**79**	M	2016	2
**28**	M	1999	18	**80**	F	2015	3
**29**	M	1981	36	**81**	M	2008	10
**30**	F	2010	8	**82**	F	2009	9
**31**	M	2016	1	**83**	F	2006	12
**32**	F	2006	11	**84**	M	2014	4
**33**	M	2010	7	**85**	F	2015	3
**34**	M	2015	2	**86**	M	2006	12

**35**	F	2015	2	**87**	F	2015	3
**36**	M	2012	5	**88**	F	1979	39
**37**	F	2010	6	**89**	M	1977	41
**38**	M	2010	8	**90**	F	2016	2
**39**	F	2005	12	**91**	M	2017	1
**40**	M	1994	23	**92**	M	2014	4
**41**	M	2002	15	**93**	M	2001	17
**42**	M	2016	1	**94**	M	2013	5
**43**	M	1997	20	**95**	F	2015	3
**44**	F	1997	20	**96**	M	2006	12
**45**	F	1999	18	**97**	M	2006	12
**46**	F	2014	3	**98**	M	2008	10
**47**	M	2009	8	**99**	F	2011	7
**48**	M	2000	18	**100**	F	2012	6
**49**	F	1994	22	**101**	M	2005	13
**50**	M	2007	9	**102**	F	2000	18
**51**	F	2002	14	**103**	F	2003	15
52	F	2002	14				

F: Female, M: Male
