# Supplementary material for: The impact of rare and low-frequency genetic variants in common variable immunodeficiency (CVID)
Source: Sci Rep. 2021 Apr 15;11:8308. doi: 10.1038/s41598-021-87898-1 (PMC8050305; doi:10.1038/s41598-021-87898-1)
Supplement: Supplementary file 2 — Supplementary information 2. [file 41598_2021_87898_MOESM2_ESM.pdf]

**The Impact of Rare and Low-Frequency Genetic Variants in Common Variable Immunodeficiency (CVID)**

Atil Bisgin[1, 2*], Ozge Sonmezler[2], Ibrahim Boga[1, 2], Mustafa Yilmaz[3]

**Supplementary Table S2**. Distribution of rare variants discovered in this study. The highest population frequency reported among all databases was used for each variant. The well-known pathogenic variants that have >10% population frequency have been excluded while the pathogenic variants shown here are the ones with differential effects in different populations.

| Gene | Variant | dbSNP rsID | ACMG Class. | Population Frequency | n (allel) | Lab. MAF |
|---|---|---|---|---|---|---|
| ***CARD11*** NM_032415.7 | c.2734C>A, p.R912R | - | VUS | 0% gnomAD | 1 | 0,4854% |
| | c.2190A>G, p.T730T | 756624250 | VUS | 0.004% gnomAD (European) | 1 | 0,9709% |
| | c.1553G>A, p.R518Q | 748468563 | VUS | 0.007% gnomAD (South Asian) | 1 | 0,4854% |
| | c.3318G>A, p.K1106K | 146564762 | VUS | 0.01% ESP (European-American) | 1 | 0,4854% |
| | c.2451G>A, p.A817A | 141131560 | VUS | 0.66% gnomAD (East Asian) | 1 | 0,4854% |
| | c.3399C>T, p.R1133R | 117428786 | B | 5.94% gnomAD (East Asian) | 1 | 0,4854% |
| ***CD19*** NM_001178098.2 | c.1541G>A, p.R514H | 34763945 | B | 6.43% ESP (European-American) | 5 | 2,4272% |
| | c.981T>C, p.T327T | - | VUS | 0% gnomAD | 1 | 0,4854% |
| | c.738G>A, p.L246L | 142412075 | LB | 0.01% ESP (European-American) | 1 | 0,4854% |
| | c.13C>T, p.R5C | 556457823 | VUS | 0.09% gnomAD (South Asian) | 1 | 0,4854% |
| | c.381G>A, p.S127S | 142342927 | VUS | 0.80% gnomAD (European) | 2 | 0,9709% |
| ***CD81*** NM_004356.4 | c.128A>G, p.N43S | 775127527 | VUS | 0.01% gnomAD (Ash. Jew.) | 1 | 0,4854% |
| | c.159C>T, p.P53P | 200365372 | VUS | 0.03% gnomAD (South Asian) | 1 | 0,4854% |
| | c.414T>C, p.D138D | 143644902 | LB | 0.35% ESP (European-American) | 1 | 0,4854% |
| ***CR2*** NM_001006658.3 | c.1678C>A, p.P560T | - | VUS | 0% gnomAD | 1 | 0,4854% |
| | c.896G>A, p.S299N | - | VUS | 0% gnomAD | 1 | 0,4854% |
| | c.221C>A, p.T74N | - | VUS | 0% gnomAD | 1 | 0,4854% |
| | c.776C>T, p.A259V | - | VUS | 0% gnomAD | 1 | 0,4854% |
| | c.2967G>A, p.M989I | 146465618 | VUS | 0.22% gnomAD (Ash. Jew.) | 1 | 0,4854% |
| | c.970C>T, p.R324C | 147393483 | VUS | 0.31% gnomAD (Ash. Jew.) | 1 | 0,4854% |
| | c.2747C>T, p.T916I | 61754517 | LB | 0.36% gnomAD (South Asian) | 1 | 0,4854% |
| | c.2445A>G, p.G815G | 141282665 | LB | 0.40% gnomAD (South Asian) | 1 | 0,4854% |
| | c.3033G>C, p.Q1011H | 200182370 | VUS | 0.42% gnomAD (South Asian) | 1 | 0,4854% |
| | c.2006A>G, p.H669R | 139230275 | VUS | 0.44% gnomAD (Ash. Jew.) | 2 | 0,9709% |
| | c.2611G>T, p.V871L | 144572703 | LB | 0.67% gnomAD (South Asian) | 3 | 1,4563% |
| | c.1676G>A, p.G559E | 143614333 | VUS | 0.80% gnomAD (Ash. Jew.) | 2 | 0,9709% |
| | c.624C>A, p.P208P | 61759494 | B | 1.54% gnomAD (Ash. Jew.) | 4 | 1,9417% |
| | c.1617C>T, p.T539T | 34349246 | B | 3.28% gnomAD (African) | 1 | 0,9709% |
| | c.3154A>G, p.I1052V | 17618 | B | 9.67% ESP (African-American) | 12 | 5,8252% |
| ***CTLA4*** NM_005214.5 | c.254G>A, p.C85Y | - | VUS | 0% gnomAD | 1 | 0,4854% |
| ***CXCR4*** NM_003467.3 | c.783C>T, p.I261I | 148279552 | LB | 0.40% ESP (European-American) | 1 | 0,4854% |
| | c.861C>T, p.T287T | 535778934 | LB | 0.02% (1000 Genomes) | 1 | 0,4854% |
| ***GATA2*** NM_032638.5 | c.189C>T, p.P63P | 763735447 | LB | 0.01% gnomAD (Latino) | 1 | 0,4854% |
| | c.1185T>C, p.T395T | 771739064 | LB | 0.07% gnomAD (Latino) | 1 | 0,4854% |
| | c.481C>G, p.P161A | 34799090 | LB | 1.58% gnomAD (Ash. Jew.) | 1 | 0,4854% |

| | | | | | | |
|---|---|---|---|---|---|---|
| | c.1233G>A, p.A411A | 34172218 | B | 6.14% gnomAD (Ash. Jew.) | 8 | 3,8835% |
| | c.564G>C, p.T188T | 34870876 | B | 6.37% gnomAD (European) | 4 | 1,9417% |
| ***ICOS*** NM_012092.4 | c.451G>C, p.V151L | 76778263 | LP | 0.73% ESP (African-American) | 1 | 0,4854% |
| ***IRF2BP2*** NM_182972.2 | c.562C>T, p.L188L | 200463649 | LB | 0% gnomAD (Latino) | 1 | 0,4854% |
| | c.352C>T, p.P118S | 148187914 | VUS | 4.83% gnomAD (Ash. Jew.) | 9 | 4,3689% |
| | c.493C>T, p.L165L | 778752231 | VUS | 0.01% gnomAD (South Asian) | 1 | 0,4854% |
| | c.1311C>T, p.G437G | 376700451 | VUS | 0.17% gnomAD (Ash. Jew.) | 1 | 0,4854% |
| | c.597G>A, p.L199L | 759578860 | LB | 0.32% gnomAD (European) | 2 | 1,4563% |
| | c.293_295dupAGC, p.Q98dup | 371994015 | B | 8.81% ESP (African-American) | 6 | 2,9126% |
| ***MOGS*** NM_006302.3 | c.1838G>A, p.R613Q | 142032474 | B | 1.62% gnomAD (Ash. Jew.) | 2 | 0,9709% |
| | c.184G>A, p.V62M | 79181168 | B | 5.90% gnomAD (European) | 6 | 2,9126% |
| | c.1403G>A, p.R468Q | 1221388589 | VUS | ≤0.001% gnomAD (European) | 1 | 0,4854% |
| | c.65C>A, p.A22E | 753961807 | VUS | 0.009% gnomAD (Latino) | 1 | 0,4854% |
| | c.1484G>A, p.R495Q | 34075781 | VUS | 0.06% gnomAD (South Asian) | 1 | 0,4854% |
| | c.881C>T, p.P294L | 184209905 | B | 1.05% gnomAD (African) | 1 | 0,4854% |
| | c.2017G>A, p.V673I | 114933392 | B | 2.45% gnomAD (Ash. Jew.) | 4 | 1,9417% |
| | c.1245C>G, p.I415M | 34838944 | B | 3.20% gnomAD (African) | 1 | 0,4854% |
| | c.2353G>A, p.G785S | 35533773 | B | 4.63% gnomAD (African) | 2 | 0,9709% |
| | c.2032C>T, p.R678W | 13405869 | B | 8.22% gnomAD (African) | 1 | 0,4854% |
| ***MS4A1*** NM_021950.3 | c.462T>C, p.F154F | 1390571456 | LB | ≤ 0.001% gnomAD (European) | 1 | 0,4854% |
| | c.328A>G, p.K110E | 201361363 | VUS | 0.03% gnomAD (European) | 1 | 0,4854% |
| ***NFKB1*** NM_003998.4 | c.2439T>C, p.A813A | 771364677 | LB | 0.13% gnomAD (Ash. Jew.) | 1 | 0,4854% |
| | c.1519A>G, p.M507V | 4648072 | VUS | 4.44% gnomAD (African) | 3 | 1,4563% |
| | c.682T>C, p.F228L | 81361488 | VUS | 0.01% gnomAD (South Asian) | 1 | 0,4854% |
| | c.1799A>C, p.E600A | 55661548 | VUS | 0.50% gnomAD (East Asian) | 1 | 0,4854% |
| | c.-8+9145A>G, - | 188317269 | VUS | 0.78% gnomAD (European) | 1 | 0,4854% |
| | c.1736G>A, p.R579K | 4648086 | VUS | 0.92% gnomAD (South Asian) | 1 | 0,4854% |
| | c.1050C>T, p.Y350Y | 4648039 | VUS | 2.45% ESP (European-American) | 2 | 0,9709% |
| | c.1755G>A, p.T585T | 4648093 | B | 8.17% ESP (African-American) | 4 | 1,9417% |
| ***NFKB2*** NM_001077494.3 | c.2678G>A, p.G893E | - | VUS | 0% gnomAD | 1 | 0,4854% |
| | c.165T>C, p.Y55Y | 755103820 | LB | 0.01% gnomAD (East Asian) | 1 | 0,4854% |
| | c.2072-3C>T, - | 201550645 | VUS | 0.35% gnomAD (Ash. Jew.) | 2 | 1,4563% |
| | c.2239C>T, p.L747L | 11191279 | LB | 1.62% gnomAD (European) | 1 | 0,4854% |
| | c.1212G>T, p.G404G | 55847683 | VUS | 3.87% gnomAD (East Asian) | 1 | 0,4854% |
| | c.2094C>T, p.N698N | 11574851 | B | 5.39% gnomAD (East Asian) | 12 | 5,8252% |
| ***PLCG2*** NM_002661.5 | c.1215C>A, p.I405I | 46020068 | VUS | 0.003% gnomAD (European) | 1 | 0,4854% |
| | c.1146T>C, p.F382F | 138637229 | LP | 2.03% gnomAD (Ash. Jew.) | 1 | 0,4854% |
| | c.2225G>A, p.R742H | 756480279 | VUS | 0.004% gnomAD (African) | 1 | 0,4854% |
| | c.1559A>G, p.D520G | 201391996 | VUS | 0.006% gnomAD (European) | 1 | 0,4854% |
| | c.2393A>G, p.N798S | 117077093 | VUS | 0.17% gnomAD (South Asian) | 1 | 0,4854% |
| | c.987G>A, p.T329T | 200506549 | LB | 0.27% gnomAD (Ash. Jew.) | 2 | 0,9709% |
| | c.923C>T, p.A308V | 199636472 | VUS | 0.57% gnomAD (Ash. Jew.) | 2 | 0,9709% |
| | c.1107C>T, p.V369V | 201652976 | LB | 0.65% gnomAD (South Asian) | 1 | 0,4854% |
| | c.1565C>G, p.P522R | 72824905 | LB | 0.89% ESP (European-American) | 4 | 1,9417% |
| | c.540C>G, p.A180A | 150276286 | LB | 0.96% ESP (European-American) | 3 | 1,4563% |
| | c.82A>T, p.M28L | 61749044 | LB | 2.11% gnomAD (South Asian) | 3 | 1,4563% |
| | c.2011A>G, p.I671V | 150833842 | LB | 2.49% gnomAD (South Asian) | 1 | 0,4854% |
| | c.1258G>A, p.A420T | 201490178 | LB | 2.91% gnomAD (South Asian) | 1 | 0,4854% |
| | c.731A>G, p.H244R | 11548656 | LB | 3.78% gnomAD (European) | 2 | 0,9709% |
| | c.770A>T, p.H257L | 45443101 | LB | 3.87% gnomAD (European) | 4 | 1,9417% |

| | | | | | | |
|---|---|---|---|---|---|---|
| | c.1188C>G, p.T396T | 13333716 | B | 8.06% gnomAD (African) | 9 | 4,8544% |
| ***TNFRSF13B*** NM_012452.3 | c.246_265delCCTGAGGG ACTGCATCAGCT, p.L83fs*9 | 1567652308 | LP | ≤ 0.001% gnomAD (European) | 1 | 0,4854% |
| | c.571G>A, p.D191N | 753867822 | VUS | 0.005% gnomAD (European) | 1 | |
| | c.431C>G, p.S144* | 104894650 | P | 0.007% gnomAD (European) | 1 | 0,4854% |
| | c.579C>A, p.C193* | 72553885 | P | 0.009% gnomAD (European) | 1 | 0,9709% |
| | c.577T>C, p.C193R | 764125338 | VUS | 0.01% gnomAD (European) | 1 | 0,9709% |
| | c.452C>T, p.P151L | 200037919 | VUS | 0.03% gnomAD (East Asian) | 1 | 0,4854% |
| | c.58C>T, p.R20C | 200013015 | VUS | 0.03% gnomAD (East Asian) | 1 | 0,4854% |
| | c.659T>C, p.V220A | 56063729 | LB | 3.58% gnomAD (Ash. Jew.) | 6 | 0,4854% |
| ***TNFRSF13C*** NM_052945.4 | c.62C>G, p.P21R | 77874543 | B | 7.90% gnomAD (European) | 10 | 2,9126% |
| | c.191_192delGCinsTT, p.G64V | 1556157858 | LP | 0% gnomAD | 5 | 6,3107% |
| | c.475C>T, p.H159Y | 61756766 | VUS | 0.71% ESP (European-American) | 1 | 2,4272% |
| ***TNFSF12*** NM_172089.4 | c.75G>T, p.A25A | 764664835 | VUS | 0.02% gnomAD (South Asian) | 1 | 0,4854% |
| ***TRNT1*** NM_182916.3 | c.686G>C, p.G229A | 773514953 | VUS | 0.003% gnomAD (European) | 1 | 0,4854% |
| | c.494C>G, p.T165S | - | VUS | 0% gnomAD | 1 | 0,4854% |
| | c.555A>C, p.G185G | - | LB | 0% gnomAD | 1 | 0,4854% |
| | c.1234C>T, p.R412* | 372367989 | VUS | 0.01% gnomAD (Latino) | 1 | 0,4854% |
| | c.133C>T, p.L45L | 75033443 | B | 8.84% gnomAD (African) | 2 | 0,4854% |
| ***TTC37*** NM_014639.4 | c.3670C>A, p.L1224M | 778799613 | VUS | 0.001% gnomAD (European) | 1 | 0,9709% |
| | c.4055A>G, p.N1352S | - | VUS | 0% gnomAD | 1 | 0,4854% |
| | c.4561A>T, p.T1521S | - | VUS | 0% gnomAD | 1 | 0,4854% |
| | c.2974C>T, p.H992Y | - | VUS | 0% gnomAD | 1 | 0,4854% |
| | c.3521G>C, p.R1174P | 149287861 | VUS | 0.01% gnomAD (European) | 1 | 0,4854% |
| | c.4379A>G, p.N1460S | 372709681 | LB | 0.06% gnomAD (African) | 1 | 0,4854% |
| | c.3603A>G, p.R1201R | 376083373 | LB | 0.18% gnomAD (Ash. Jew.) | 1 | 0,4854% |
| | c.3808C>G, p.P1270A | 146627706 | B | 0.32% gnomAD (South Asian) | 2 | 0,4854% |
| | c.4187A>G, p.N1396S | 116690692 | B | 0.72% gnomAD (European) | 2 | 0,9709% |

ACMG: American College of Genetics and Genomics, gnomAD: The Genome Aggregation Database, ESP: Exome Sequencing Project, dbSNP: The Single Nucleotide Polymorphism Database, -: novel variation, P: Pathogenic, LP: Likely Pathogenic, VUS: Variant of uncertain significance, LB: Likely benign, B: Benign, MAF: Minor Allel Frequency.
