# Supplementary material for: The impact of rare and low-frequency genetic variants in common variable immunodeficiency (CVID)
Source: Sci Rep. 2021 Apr 15;11:8308. doi: 10.1038/s41598-021-87898-1 (PMC8050305; doi:10.1038/s41598-021-87898-1)
Supplement: Supplementary file 3 — Supplementary information 3. [file 41598_2021_87898_MOESM3_ESM.pdf]

The Impact of Rare and Low-Frequency Genetic Variants in Common Variable Immunodeficiency (CVID)

Atil Bisgin[1, 2*], Ozge Sonmezler[2], Ibrahim Boga[1, 2], Mustafa Yilmaz[3]

Supplementary Table S3. Details of detected variants in 103 CVID patients.

Patient	Gene	Variation		ACMG Class.
**P1**	*TNFRSF13B*	c.659T>C p.V220A	*Heterozygote*	Likely Benign
	CD19	c.1541G>A p.R514H	*Heterozygote*	Benign
	CR2	c.1617C>T p.T539T	***Homozygote***	Benign
	TTC37	c.4187A>G p.N1396S	*Heterozygote*	Benign
**P2**	*TNFRSF13B*	c.579C>A p.C193*	*Heterozygote*	Pathogenic
	NFKB1	c.1799A>C p.E600A	*Heterozygote*	VUS
	GATA2	c.1233G>A p.A411A	*Heterozygote*	Benign
**P3**	*TNFRSF13B*	c.571G>A p.D191N	*Heterozygote*	VUS
	CR2	c.2611G>T p.V871L	*Heterozygote*	Likely Benign
**P4**	*PLCG2*	c.2393A>G p.N798S	*Heterozygote*	VUS
	TNFRSF13B	c.659T>C p.V220A	*Heterozygote*	Likely Benign
	CR2	c.3154A>G p.I1052V	*Heterozygote*	Benign
**P5**	*MOGS*	c.184G>A p.V62M	*Heterozygote*	Benign
**P6**	*NFKB1*	c.682T>C p.F228L	*Heterozygote*	VUS
	PLCG2	c.1565C>G p.P522R	*Heterozygote*	Likely Benign
**P7**	*TNFRSF13B*	c.246_265delCCTGAGGGACTGCATCAGCT p.L83fs*9	*Heterozygote*	Likely Pathogenic
	IRF2BP2	c.293_295dupAGC p.Q98dup	*Heterozygote*	Benign
**P8**	*TNFRSF13C*	c.191_192delGCinsTT p.G64V	*Heterozygote*	Likely Pathogenic
	TRNT1	c.686G>C p.G229A	*Heterozygote*	VUS
	MOGS	c.65C>A p.A22E	*Heterozygote*	VUS
**P9**	*TNFRSF13C*	c.191_192delGCinsTT p.G64V	*Heterozygote*	Likely Pathogenic
	CTLA4	c.254G>A p.C85Y	*Heterozygote*	VUS
	PLCG2	c.1146T>C p.F382F	*Heterozygote*	Likely Pathogenic
	PLCG2	c.731A>G p.H244R	*Heterozygote*	Likely Pathogenic
	GATA2	c.1233G>A p.A411A	*Heterozygote*	Benign
**P10**	*CR2*	c.3154A>G p.I1052V	*Heterozygote*	Benign
**P11**	*TNFSF12*	c.75G>T p.A25A	*Heterozygote*	VUS
	MOGS	c.184G>A p.V62M	*Heterozygote*	Benign
**P12**	*IRF2BP2*	c.352C>T p.P118S	*Heterozygote*	VUS
	NFKB2	c.2094C>T p.N698N	*Heterozygote*	Benign
	MOGS	c.184G>A p.V62M	*Heterozygote*	Benign
**P13**	*PLCG2*	c.2011A>G p.I671V	*Heterozygote*	Likely Benign
**P14**	*IRF2BP2*	c.352C>T p.P118S	*Heterozygote*	VUS
	NFKB2	c.2094C>T p.N698N	*Heterozygote*	Benign
**P15**	*PLCG2*	c.1188C>G p.T396T	*Heterozygote*	Benign
**P16**	*CD19*	c.381G>A p.S127S	*Heterozygote*	VUS

	IRF2BP2	c.352C>T p.P118S	*Heterozygote*	VUS
**P17**	*CR2*	c.221C>A p.T74N	*Heterozygote*	VUS
**P18**	*NFKB1*	c.1519A>G p.M507V	*Heterozygote*	VUS
	MOGS	c.184G>A p.V62M	*Heterozygote*	Benign
**P19**	*PLCG2*	c.2225G>A p.R742H	*Heterozygote*	VUS
	TTC37	c.4187A>G p.N1396S	*Heterozygote*	Benign
**P20**	*MS4A1*	c.462T>C p.F154F	*Heterozygote*	Likely Benign
	MOGS	c.2017G>A p.V673I	*Heterozygote*	Benign
	CD19	c.1541G>A p.R514H	*Heterozygote*	Benign
**P21**	*TTC37*	c.2974C>T p.H992Y	*Heterozygote*	VUS
	CXCR4	c.783C>T p.I261I	*Heterozygote*	Likely Benign
	CR2	c.3154A>G p.I1052V	*Heterozygote*	Benign
**P22**	*MOGS*	c.1838G>A p.R613Q	*Heterozygote*	Benign
**P23**	*PLCG2*	c.1559A>G p.D520G	*Heterozygote*	VUS
	GATA2	c.564G>C p.T188T	*Heterozygote*	Benign
**P24**	*CR2*	c.776C>T p.A259V	*Heterozygote*	VUS
**P25**	*TNFRSF13C*	c.475C>T p.H159Y	*Heterozygote*	VUS
	TNFRSF13C	c.62C>G p.P21R	*Heterozygote*	Benign
**P26**	*GATA2*	c.1233G>A p.A411A	*Heterozygote*	Benign
**P27**	*IRF2BP2*	c.562C>T p.L188L	*Heterozygote*	Likely Benign
	CARD11	c.2190A>G p.T730T	***Homozygote***	VUS
	IRF2BP2	c.493C>T p.L165L	*Heterozygote*	VUS
	IRF2BP2	c.293_295dupAGC p.Q98dup	*Heterozygote*	Benign
**P28**	*TTC37*	c.4561A>T p.T1521S	*Heterozygote*	VUS
	NFKB1	c.2439T>C p.A813A	*Heterozygote*	Likely Benign
**P29**	*TTC37*	c.3603A>G p.R1201R	*Heterozygote*	Likely Benign
	PLCG2	c.770A>T p.H257L	*Heterozygote*	Likely Benign
**P30**	*PLCG2*	c.1107C>T p.V369V	*Heterozygote*	Likely Benign
	MOGS	c.1245C>G p.I415M	*Heterozygote*	Benign
	MOGS	c.2353G>A p.G785S	*Heterozygote*	Benign
**P31**	*CXCR4*	c.861C>T p.T287T	*Heterozygote*	Likely Benign
	TNFRSF13B	c.659T>C p.V220A	*Heterozygote*	Likely Benign
	NFKB2	c.2094C>T p.N698N	*Heterozygote*	Benign
	TNFRSF13C	c.62C>G p.P21R	*Heterozygote*	Benign
**P32**	*PLCG2*	c.1215C>A p.I405I	*Heterozygote*	VUS
	MOGS	c.2017G>A p.V673I	*Heterozygote*	Benign
	GATA2	c.1233G>A p.A411A	*Heterozygote*	Benign
**P33**	*CD19*	c.981T>C p.T327T	*Heterozygote*	VUS
**P34**	*MOGS*	c.2017G>A p.V673I	*Heterozygote*	Benign
	IRF2BP2	c.352C>T p.P118S	*Heterozygote*	VUS
	NFKB2	c.2094C>T p.N698N	*Heterozygote*	Benign
	GATA2	c.564G>C p.T188T	*Heterozygote*	Benign
**P35**	*CARD11*	c.3318G>A p.K1106K	*Heterozygote*	VUS
	CR2	c.624C>A p.P208P	*Heterozygote*	Benign
**P36**	*CARD11*	c.1553G>A p.R518Q	*Heterozygote*	VUS
	IRF2BP2	c.597G>A p.L199L	***Homozygote***	Likely Benign

	TNFRSF13B	c.659T>C p.V220A	*Heterozygote*	Likely Benign
	CR2	c.3154A>G p.I1052V	*Heterozygote*	Benign
**P37**	*NFKB2*	c.165T>C p.Y55Y	*Heterozygote*	Likely Benign
	GATA2	c.189C>T p.P63P	*Heterozygote*	Likely Benign
	PLCG2	c.923C>T p.A308V	*Heterozygote*	VUS
	CARD11	c.2451G>A p.A817A	*Heterozygote*	VUS
**P38**	*CR2*	c.1676G>A p.G559E	*Heterozygote*	VUS
**P39**	*NFKB2*	c.2094C>T p.N698N	*Heterozygote*	Benign
**P40**	*PLCG2*	c.770A>T p.H257L	*Heterozygote*	Likely Benign
	NFKB2	c.1212G>T p.G404G	*Heterozygote*	VUS
	IRF2BP2	c.352C>T p.P118S	*Heterozygote*	VUS
	CD19	c.1541G>A p.R514H	*Heterozygote*	Benign
	MOGS	c.2032C>T p.R678W	*Heterozygote*	Benign
**P41**	*GATA2*	c.481C>G p.P161A	*Heterozygote*	Likely Benign
**P42**	*TTC37*	c.3670C>A p.L1224M	*Heterozygote*	VUS
	TTC37	c.3808C>G p.P1270A	*Heterozygote*	Benign
	TNFRSF13C	c.62C>G p.P21R	***Homozygote***	Benign
	PLCG2	c.1188C>G p.T396T	*Heterozygote*	Benign
	CR2	c.3154A>G p.I1052V	*Heterozygote*	Benign
**P43**	*No Rare Variants Detected*			
**P44**	*No Rare Variants Detected*			
**P45**	*GATA2*	c.1185T>C p.T395T	*Heterozygote*	Likely Benign
**P46**	*NFKB2*	c.2094C>T p.N698N	*Heterozygote*	Benign
	CD19	c.1541G>A p.R514H	*Heterozygote*	Benign
**P47**	*TTC37*	c.3521G>C p.R1174P	*Heterozygote*	VUS
	MOGS	c.1838G>A p.R613Q	*Heterozygote*	Benign
	MOGS	c.2353G>A p.G785S	*Heterozygote*	Benign
**P48**	*PLCG2*	c.923C>T p.A308V	*Heterozygote*	VUS
	TNFRSF13C	c.62C>G p.P21R	*Heterozygote*	Benign
**P49**	*NFKB2*	c.2094C>T p.N698N	***Homozygote***	Benign
**P50**	*NFKB2*	c.2072-3C>T -	*Heterozygote*	VUS
	GATA2	c.564G>C p.T188T	*Heterozygote*	Benign
**P51**	*PLCG2*	c.770A>T p.H257L	*Heterozygote*	Likely Benign
	PLCG2	c.1188C>G p.T396T	*Heterozygote*	Benign
	CR2	c.3154A>G p.I1052V	*Heterozygote*	Benign
**P52**	*NFKB1*	c.1736G>A p.R579K	*Heterozygote*	VUS
	IRF2BP2	c.293_295dupAGC p.Q98dup	*Heterozygote*	Benign
**P53**	*CR2*	c.2006A>G p.H669R	*Heterozygote*	VUS
**P54**	*CR2*	c.2747C>T p.T916I	*Heterozygote*	Likely Benign
	PLCG2	c.540C>G p.A180A	*Heterozygote*	Likely Benign
	MOGS	c.184G>A p.V62M	*Heterozygote*	Benign
**P55**	*TRNT1*	c.555A>C p.G185G	*Heterozygote*	Likely Benign
	TNFRSF13C	c.62C>G p.P21R	*Heterozygote*	Benign
**P56**	*IRF2BP2*	c.352C>T p.P118S	*Heterozygote*	VUS
**P57**	*PLCG2*	c.987G>A p.T329T	*Heterozygote*	Likely Benign
	CR2	c.2006A>G p.H669R	*Heterozygote*	VUS

	NFKB1	c.1755G>A p.T585T	*Heterozygote*	Benign
	TRNT1	c.133C>T p.L45L	*Heterozygote*	Benign
**P58**	*NFKB1*	c.1050C>T p.Y350Y	*Heterozygote*	VUS
	CARD11	c.3399C>T p.R1133R	*Heterozygote*	Benign
	GATA2	c.1233G>A p.A411A	*Heterozygote*	Benign
**P59**	*No Rare Variants Detected*			
**P60**	*CD81*	c.128A>G p.N43S	*Heterozygote*	VUS
	CD19	c.13C>T p.R5C	*Heterozygote*	VUS
	CR2	c.3033G>C p.Q1011H	*Heterozygote*	VUS
	NFKB1	c.-8+9145A>G -	*Heterozygote*	VUS
	PLCG2	c.1188C>G p.T396T	*Heterozygote*	Benign
	CR2	c.3154A>G p.I1052V	*Heterozygote*	Benign
**P61**	*CR2*	c.1678C>A p.P560T	*Heterozygote*	VUS
	IRF2BP2	c.352C>T p.P118S	*Heterozygote*	VUS
**P62**	*PLCG2*	c.987G>A p.T329T	*Heterozygote*	Likely Benign
	CR2	c.3154A>G p.I1052V	*Heterozygote*	Benign
**P63**	*TNFRSF13B*	c.659T>C p.V220A	*Heterozygote*	Likely Benign
**P64**	*No Rare Variants Detected*			
**P65**	*No Rare Variants Detected*			
**P66**	*CD19*	c.738G>A p.L246L	*Heterozygote*	Likely Benign
	TNFRSF13C	c.62C>G p.P21R	***Homozygote***	Benign
	NFKB1	c.1755G>A p.T585T	*Heterozygote*	Benign
**P67**	*TTC37*	c.3808C>G p.P1270A	*Heterozygote*	Benign
	CR2	c.1676G>A p.G559E	*Heterozygote*	VUS
	PLCG2	c.82A>T p.M28L	*Heterozygote*	Likely Benign
**P68**	*No Rare Variants Detected*			
**P69**	*NFKB2*	c.2094C>T p.N698N	***Homozygote***	Benign
	IRF2BP2	c.293_295dupAGC p.Q98dup	*Heterozygote*	Benign
**P70**	*PLCG2*	c.770A>T p.H257L	*Heterozygote*	Likely Benign
	TNFRSF13C	c.62C>G p.P21R	***Homozygote***	Benign
**P71**	*TNFRSF13B*	c.452C>T p.P151L	*Heterozygote*	VUS
	NFKB2	c.2094C>T p.N698N	*Heterozygote*	Benign
**P72**	*MOGS*	c.1484G>A p.R495Q	*Heterozygote*	VUS
	NFKB1	c.1755G>A p.T585T	*Heterozygote*	Benign
**P73**	*No Rare Variants Detected*			
**P74**	*MS4A1*	c.328A>G p.K110E	*Heterozygote*	VUS
	TNFRSF13B	c.659T>C p.V220A	*Heterozygote*	Likely Benign
	CD19	c.1541G>A p.R514H	*Heterozygote*	Benign
	PLCG2	c.1188C>G p.T396T	***Homozygote***	Benign
	NFKB1	c.1755G>A p.T585T	*Heterozygote*	Benign
**P75**	*TTC37*	c.4379A>G p.N1460S	*Heterozygote*	Likely Benign
	MOGS	c.2017G>A p.V673I	*Heterozygote*	Benign
**P76**	*MOGS*	c.1403G>A p.R468Q	*Heterozygote*	VUS
	PLCG2	c.540C>G p.A180A	*Heterozygote*	Likely Benign
	NFKB2	c.2239C>T p.L747L	*Heterozygote*	Likely Benign
	CR2	c.3154A>G p.I1052V	*Heterozygote*	Benign

**P77**	*CD81*	c.159C>T p.P53P	*Heterozygote*	VUS
**P78**	*TRNT1*	c.494C>G p.T165S	*Heterozygote*	VUS
	CR2	c.3154A>G p.I1052V	*Heterozygote*	Benign
**P79**	*CR2*	c.624C>A p.P208P	*Heterozygote*	Benign
**P80**	*No Rare Variants Detected*			
**P81**	*No Rare Variants Detected*			
**P82**	*CD81*	c.414T>C p.D138D	*Heterozygote*	Likely Benign
	PLCG2	c.1565C>G p.P522R	*Heterozygote*	Likely Benign
**P83**	*PLCG2*	c.1188C>G p.T396T	*Heterozygote*	Benign
**P84**	*TNFRSF13B*	c.58C>T p.R20C	*Heterozygote*	VUS
	CR2	c.3154A>G p.I1052V	*Heterozygote*	Benign
**P85**	*PLCG2*	c.540C>G p.A180A	*Heterozygote*	Likely Benign
	CR2	c.624C>A p.P208P	*Heterozygote*	Benign
	GATA2	c.1233G>A p.A411A	*Heterozygote*	Benign
	GATA2	c.564G>C p.T188T	*Heterozygote*	Benign
**P86**	*PLCG2*	c.1258G>A p.A420T	*Heterozygote*	Likely Benign
	MOGS	c.184G>A p.V62M	*Heterozygote*	Benign
	TNFRSF13C	c.62C>G p.P21R	*Heterozygote*	Benign
**P87**	*TNFRSF13B*	c.431C>G p.S144*	***Homozygote***	Pathogenic
	TRNT1	c.1234C>T p.R412*	*Heterozygote*	VUS
	TNFRSF13B	c.577T>C p.C193R	***Homozygote***	VUS
**P88**	*CR2*	c.896G>A p.S299N	*Heterozygote*	VUS
	IRF2BP2	c.597G>A p.L199L	*Heterozygote*	Likely Benign
	IRF2BP2	c.352C>T p.P118S	*Heterozygote*	VUS
	NFKB2	c.2094C>T p.N698N	*Heterozygote*	Benign
**P89**	*No Rare Variants Detected*			
**P90**	*No Rare Variants Detected*			
**P91**	*CR2*	c.624C>A p.P208P	*Heterozygote*	Benign
	PLCG2	c.1188C>G p.T396T	*Heterozygote*	Benign
**P92**	*CR2*	c.2445A>G p.G815G	*Heterozygote*	Likely Benign
	GATA2	c.1233G>A p.A411A	*Heterozygote*	Benign
	TNFRSF13C	c.62C>G p.P21R	*Heterozygote*	Benign
	IRF2BP2	c.293_295dupAGC p.Q98dup	*Heterozygote*	Benign
**P93**	*No Rare Variants Detected*			
**P94**	*TNFRSF13C*	c.191_192delGCinsTT p.G64V	*Heterozygote*	Likely Pathogenic
	CARD11	c.2734C>A p.R912R	*Heterozygote*	VUS
	IRF2BP2	c.896T>C p.V299A	*Heterozygote*	VUS
	PLCG2	c.1188C>G p.T396T	*Heterozygote*	Benign
**P95**	*TTC37*	c.4055A>G p.N1352S	*Heterozygote*	VUS
	NFKB2	c.2072-3C>T -	***Homozygote***	VUS
	CD19	c.381G>A p.S127S	*Heterozygote*	VUS
	NFKB1	c.1050C>T p.Y350Y	*Heterozygote*	VUS
**P96**	*TNFRSF13C*	c.191_192delGCinsTT p.G64V	*Heterozygote*	Likely Pathogenic
	CR2	c.2611G>T p.V871L	*Heterozygote*	Likely Benign
	PLCG2	c.1565C>G p.P522R	*Heterozygote*	Likely Benign
	PLCG2	c.82A>T p.M28L	*Heterozygote*	Likely Benign

	NFKB1	c.1519A>G p.M507V	*Heterozygote*	VUS
**P97**	*No Rare Variants Detected*			
**P98**	*TNFRSF13C*	c.191_192delGCinsTT p.G64V	*Heterozygote*	Likely Pathogenic
	CR2	c.2611G>T p.V871L	*Heterozygote*	Likely Benign
	PLCG2	c.1565C>G p.P522R	*Heterozygote*	Likely Benign
	PLCG2	c.82A>T p.M28L	*Heterozygote*	Likely Benign
	NFKB1	c.1519A>G p.M507V	*Heterozygote*	VUS
	TRNT1	c.133C>T p.L45L	*Heterozygote*	Benign
**P99**	*NFKB2*	c.2678G>A p.G893E	*Heterozygote*	VUS
	IRF2BP2	c.1311C>T p.G437G	*Heterozygote*	VUS
**P100**	*CR2*	c.2967G>A p.M989I	*Heterozygote*	VUS
	MOGS	c.881C>T p.P294L	*Heterozygote*	Benign
	TNFRSF13C	c.62C>G p.P21R	*Heterozygote*	Benign
	CR2	c.3154A>G p.I1052V	*Heterozygote*	Benign
**P101**	*ICOS*	c.451G>C p.V151L	*Heterozygote*	Likely Pathogenic
**P102**	*IRF2BP2*	c.352C>T p.P118S	*Heterozygote*	VUS
	GATA2	c.1233G>A p.A411A	*Heterozygote*	Benign
	PLCG2	c.1188C>G p.T396T	*Heterozygote*	Benign
**P103**	*CR2*	c.970C>T p.R324C	*Heterozygote*	VUS
	PLCG2	c.731A>G p.H244R	*Heterozygote*	Likely Pathogenic

CVID: Common Variable Immunodeficiency, ACMG: American College of Genetics and Genomics, gnomAD: The Genome Aggregation Database, ESP: Exome Sequencing Project, VUS: Variant of uncertain significance.
